# GABA Neurons in the Ventral Tegmental Area Responding to Peripheral Sensory Input

**DOI:** 10.1371/journal.pone.0051507

**Published:** 2012-12-10

**Authors:** Chang-Liang Liu, Ming Gao, Guo-Zhang Jin, Xuechu Zhen

**Affiliations:** 1 Department of Pharmacology, State Key Laboratory of Drug Research, Institute of Materia Medica, Shanghai Institute for Biological Sciences, Chinese Academy of Sciences, Shanghai, China; 2 Department of Pharmacology, Soochow University, College of Pharmaceutical Sciences, Suzhou, China; Georgia Health Sciences University, United States of America

## Abstract

Dopamine (DA) neurons in the ventral tegmental area (VTA) not only participate in reward processing, but also respond to aversive stimuli. Although GABA neurons in this area are actively involved in regulating the firing of DA neurons, few data exist concerning the responses of these neurons to aversive sensory input. In this study, by employing extracellular single-unit recording and spectral analysis techniques in paralyzed and ventilated rats, we found that the firing pattern in 44% (47 of 106) of GABA cells in the VTA was sensitive to the sensory input produced by the ventilation, showing a significant ventilation-associated oscillation in the power spectra. Detailed studies revealed that most ventilation-sensitive GABA neurons (38 of 47) were excited by the stimuli, whereas most ventilation-sensitive DA neurons (11 of 14) were inhibited. When the animals were under anesthesia or the sensory pathways were transected, the ventilation-associated oscillation failed to appear. Systemic administration of non-competitive N-methyl-D-aspartase (NMDA) receptor antagonist MK-801 completely disrupted the association between the firing of GABA neurons and the ventilation. Interestingly, local MK-801 injection into the VTA dramatically enhanced the sensitivity of GABA neurons to the ventilation. Our data demonstrate that both GABA and DA neurons in the VTA can be significantly modulated by sensory input produced by the ventilation, which may indicate potential functional roles of VTA in processing sensation-related input.

## Introduction

Mesocorticolimbic dopamine (DA) system, originating from the VTA and projecting to the prefrontal cortex, hippocampus, amygdale, and nucleus accumbens, plays essential roles in reward, motivational control, and modulation of cognitive functions [1], [2], [3], [4], [5]. The DA neurons in the VTA exhibit rapid and brief bursts in response to unexpected rewarding events, whereas are inhibited when the reward is omitted or has less value than expected [4], [6]. On the other hand, however, studies have also found that DA neurons in the VTA can respond to a category of events that includes but extends beyond that of rewards. Several studies have reported that salient but aversive stimuli can significantly regulate the activity of DA neurons [7], [8], [9]. In addition, DA depletion results in a decrease in orientation to visual tactile and olfactory stimuli, and conversely, DA agonists or genetic manipulations of DA signaling produce altered responses to sensory stimuli, and interfere with acquisition and expression of behaviors such as aversive conditioning [10], [11], [12], [13]. Similar sensory gating deficits are also observed from schizophrenic patients [14], a population believed to suffer from abnormalities in DA transmission. All of these evidences indicate that in addition to reward, DA neurons in the VTA also play an important role in sensory or attentional processing.

In the VTA, the DA neurons make up about 55–65% of the neurons, the rest are mainly GABA neurons. GABA neurons are fast spiking cells without detectable tyrosine hydrocylase (TH) immunoreactivity, and lie in close proximity to TH-labeled cells [15], [16]. These neurons synapse directly onto DA neurons and are believed playing essential roles in regulating DA neuronal activity. Activation of GABA neurons could remarkably suppress the activity of neighboring DA neurons, and therefore affect animal behaviors such as reward consumption [17]. In addition to modulating the activity of DA cells, GABA neurons themselves can also encode information during prediction error coding [18]. Gathering evidence suggests that the local GABA neurons in the VTA are not only an important regulator of the DA cells, but also an essential component for the functional expression of the VTA. Although significant efforts have been made to elucidate the roles of GABA neurons in VTA-associated rewarding behaviors, little is known on whether and how GABA neurons respond to non-rewarding sensory stimuli. A very recent study, however, reported that local GABA neurons in the VTA could be excited by footshock, an evidence that these neurons may also be actively involved during non-rewarding stimuli [19]. We thus aimed to observe the possible influences of non-rewarding stimuli on local VTA GABA neurons.

Since sensory input is typically attenuated in animals under anesthesia we made recordings directly from single VTA GABA and DA neurons in awake and paralyzed rats under artificial ventilation, and used spectral analysis techniques to study their firing patterns. We provide evidence that the sensory input produced by the ventilation can substantially regulate the activity of both the GABA and DA neurons in the VTA. Most GABA neurons were excited by the ventilation while most DA neurons were inhibited. The GABA neurons responded to the ventilation pulse more quickly than the DA neurons. In addition, systemic and local NMDA receptor blockade fundamentally altered the sensitivity of GABA neurons to the sensory input.

## Materials and Methods

### Ethics Statement

Male Sprageue-Dawley rats, weighting 200–250 g were purchased from Shanghai Slac Laboratory Animal Co. Ltd (Shanghai, China). The experimental protocols were approved and strictly followed by the Institutional Animal Care and Use Committees of Shanghai Institute of Materia Medica (IACUC permit number: 2010-12-ZXC-02), and were in compliance with the Guidelines for the Care and Use of Laboratory Animals (National Research Council, People’s Republic of China, 2010).

### Animal Preparations

For the experiments in awake, paralyzed rats, animals were first anesthetized with diethyl ether to allow surgical preparations. A tracheostomy was performed to permit artificial ventilation. Rats were paralyzed with gallamine triethiodide (30 mg/kg, dissolved in distilled water, Sigma, CA US), with additional doses administered via a lateral tail vein. After gallamine injection, rats were artificially ventilated (70 cycles/min, 3.0–4.0 ml/cycle) with O_2_–enriched room air via a respirator (DH-150, Zhejiang University, China). All pressure points and incision sites were infiltrated with the local anesthetic lidocaine hydrochloride (2%, Sigma).

For the lesion studies, bilateral vagotomies or spinal cord transaction or both were performed before electrophysiological recording. For experiments in anesthetized rats under artificial respiration, animals were first anesthetized with chloral hydrate (400 mg/kg, i.p., dissolved in 0.9% saline, Sinopharm Chemical Reagent Co., LTD, Shanghai) and then treated the same way as awake, paralyzed rats. Supplemental doses of chloral hydrate and gallamine triethiodide were administered via a lateral tail vein as needed. Some experiments were performed in rats under anesthesia but without artificial respiration. No gallamine triethiodide was given in this group.

### Electrophysiological Recordings

Extracellular single-unit recordings were performed using methods similar to those described previously [20]. Briefly, rats were mounted in a stereotaxic apparatus with body temperature maintained at 37.0±0.2°C by a feedback heating pad (Harvard Apparatus, US). The skull was exposed and a hole was drilled to accommodate the placement of recording electrode. Glass microelectrodes were made using a Narishige (Tokyo, Japan) electrode puller and were filled with 2 M NaCl solution containing 2% Pontamine Sky Blue dye. The tip of the electrode was between 0.3 and 1 µm with resistance between 5 and 10 MΩ. To record single-unit activity, signals were amplified (2000×) and filtered (80–10,000 Hz). The artificial ventilation pressure was measured via a pressure transducer and was recorded simultaneously with the neuronal discharge by an A/D converter (SMUP-E, Fudan University, China). The electrode was oriented, via stereotaxic coordinates, into the VTA (from lamda: 2.7–3.3 mm anterior; 0.5–0.9 mm lateral and 7.0–8.5 mm ventral). GABA neurons were identified by their rapid, non-bursting activity (>10 Hz) and the short duration action potentials (<1 ms) [16], [21]. DA neurons were identified by well established electrophysiological criteria [7], [22]. The onset of a burst was identified as two consecutive spikes with an interval of shorter than 80 ms, and the termination of a burst was defined as an interval of longer than 160 ms [23]. At the end of the experiment, the animals were killed with overdose of chloral hydrate. Brain slices were cut and the recording site was verified by Pontamine Sky Blue delivered by iontophoresis after each experiment.

### Data Analysis

Firing periodicity was analyzed using methods as described previously [20]. Briefly, rate histograms with a bin width of 50 ms were constructed based on a 2 min segment of recording. Following tapering (Tukey window function; 7 windows with 50% overlap) and removal of the linear trend, a fast Fourier transform was performed to yield spectra with a resolution of 0.039 Hz. The amplitude of a spectral peak was expressed as a proportion of total power so that the sum of all peaks equals 100. To help visualize slow changes in rate histograms, data were smoothed using the following function:
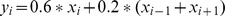
where y_i_ is the smoothed value, x_i_ is the original data, x_i−1_ and x_i+1_ are values immediately before and after x_i_ respectively. Each data series was repeatedly smoothed for 10 times. All analyses, however, were based on non-smoothed data.

Differences between groups were determined by one-way ANOVA followed by *post hoc* Tukey test. Effects of drugs before and after the application were evaluated by paired *t* test. Data sets with unequal variances were log-transformed before statistical comparison. A *p* value<0.05 was considered significant.

## Results

### Artificial Ventilation Regulates the Firing of VTA GABA Neurons

**Figure 1:**
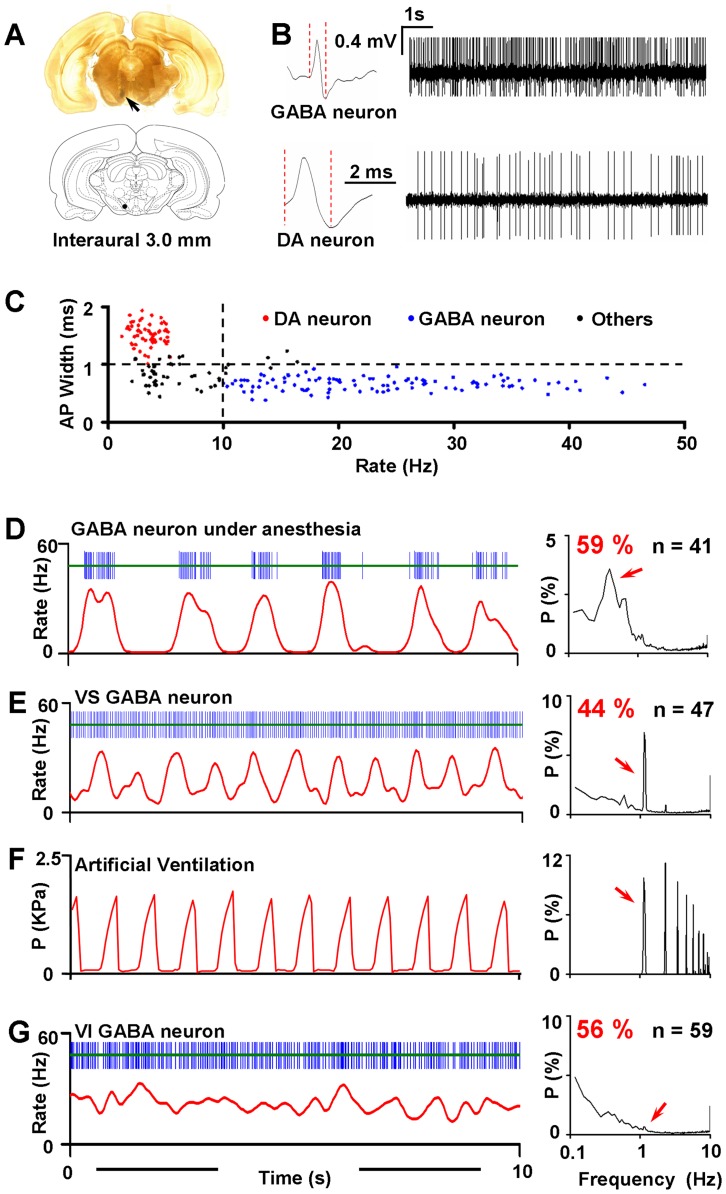
VTA GABA neurons respond to artificial ventilation. (**A**) Anatomical locations for *in vivo* single-unit recordings (arrow). Pontamine Sky Blue was delivered iontophoretically after each recording. If not mentioned, several cells were recorded from one animal, regardless of the subdivision of the VTA. (**B**) Representative wave form and firing activity from putative GABA and DA neurons. The red dashed lines indicate the definition of AP width in our study. Note the fast firing rate and short term action potential of GABA neurons compared with putative DA neurons. (**C**) Criteria for classification of neuronal types. Neurons that were not classified as typical DA or GABA neurons (black dots) were abandoned in this study. (**D**) Schematic drawing of a segment of spike trains from a GABA neuron (blue bars) showing robust slow oscillation. The neuron was recorded in the VTA from a rat under anesthesia. The corresponding 50 ms bin-width rate histogram was constructed and smoothed (red line; see methods) [20]. Slow rhythmic fluctuations can be clearly observed in the smoothed rate histogram. The right chart indicates the mean power spectrum averaged from all neurons exhibiting significant slow oscillation. The x-axis was log-transformed to emphasize oscillations in low frequencies. A prominent slow oscillation peaked at 0.39 Hz (red arrow) can be found in the spectrum. In this and the following power spectra, the power of a spectral peak was expressed as a proportion of total power so that the sum of all peaks equals 100. (**E**) Similar to A, but from a typical ventilation-sensitive GABA neuron recorded in an awake and paralyzed rat. A robust spectral peak at 1.13 Hz existed in the averaged power spectrum (red arrow) of all ventilation-sensitive neurons. (**F**) A segment of pressure signal of ventilation (left) and the corresponding power spectrum of the signal (right). Arrow indicates an oscillation at 1.13 Hz. (**G**) Similar to B, but from a neuron without detectable ventilation-associated activity. No significant peak can be found around 1.13 Hz (arrow) in the mean power spectrum of these neurons.

Single-unit recordings were made both in anesthetized and in conscious rats. In most cases, spontaneous activity of neurons was recorded while passing an electrode through the VTA. 12 electrode tracks separated by 200 µm were made in each animal and the recording sites were identified by delivering Pontamine Sky Blue after each recording (Figure 1A). The cells recorded in our study were from both the ventral and dorsal VTA and were analyzed together. Two main categories of cells were found in the VTA. The putative GABA neurons were identified by their rapid, non-bursting activity (>10 Hz) and the short duration action potentials (AP<1 ms, or half-action potential durations of <0.5 ms, measured from the start of AP to the negative trough) [16], [21]. Whereas, the putative DA neurons were identified by relatively low firing rate (<10 Hz), biphasic spike waveform with a duration of >3.0 ms, and a broad initial positive phase of >1 ms (Figure 1B and C) [7], [22]. Neurons that were not typically classified as GABA or DA cells were abandoned in this study. On average the AP width of putative GABA neurons was 0.65±0.01 ms (n = 106) while the AP width of putative DA neurons was 1.51±0.03 ms (n = 58).

Most GABA neurons recorded in anesthetized rats fired in a phasic 1.0–3.0 s ON/OFF periodic pattern [24]. After spectral analysis, a slow oscillation, residing in 0.3–1.0 Hz was usually found in their power spectra (59%; 41 of 69 cells; Figure 1C and Figure 4A). In awake and paralyzed rats, however, spectral analysis of the firing trains of GABA neurons often revealed a robust oscillation at 1.13 Hz (Figure 1D), a frequency very close to that of artificial ventilation (70 cycles/min). To confirm the coincidence, similar analyses were applied to the ventilation pressure signal. As expected, a pronounced spectral peak appeared exactly at 1.13 Hz in the power spectra (Figure 1E). These results indicate that the firing pattern of GABA neurons is associated with the mechanical stimuli generated by the artificial ventilation. However, it should be noted that not every GABA neuron exhibited significant ventilation-associated oscillation (VO). In the total 106 cells recorded (from 11 rats), 44% (47 of 106 cells) met the criterion for ventilation-sensitive (VS) cells: in their power spectra, the peak power in 1.13 Hz (P_1.13Hz_) was significantly higher than the mean power between 1.0 and 1.5 Hz (*t* test). The other neurons (56%; 59 of 106 cells), however, showed no significant rhythm in response to the ventilation, and therefore were referred to as ventilation-insensitive cells (VI; Figure 1F).

### Most VS GABA Neurons are Excited by the Ventilation

**Figure 2:**
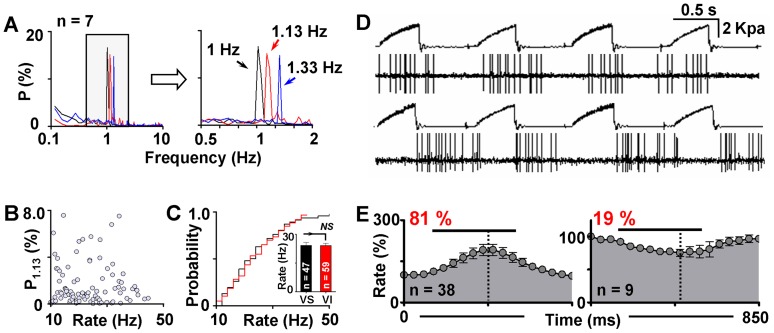
Relationships between ventilation and firing rate of GABA neurons. (**A**) Averaged and x-axis enlarged (right) power spectra showing the firing patterns of VS neurons under the ventilation of 60 (black), 70 (red), and 80 (blue) cycles/min. Corresponding peaks can be clearly observed in the power spectra of the conditions indicated. (**B**) The peak power at 1.13 Hz was plotted against the corresponding firing rate. No significant relationship can be found between the two parameters. (**C**) Mean firing rate and the cumulative firing rate distributions of VS (black) and VI (red) neurons. NS indicates not significant. (**D**) Representative raw recordings of GABA neurons exhibiting excitation (top) and inhibition (bottom) to the ventilation pulse. The ventilation pulse and the firing activity of the neuron were recorded simultaneously. (**E**) Averaged changes of the firing rate in response to the ventilation pulse (black lines). Dashed lines indicate the strongest excitation or inhibition point.

Since the firing patterns of GABA neurons in conscious animals were highly associated with artificial ventilation, we wondered whether changing the ventilation frequency could alter the VO coordinately. 7 VS GABA neurons were chosen to carry out the experiment, and three different frequencies of ventilation (60, 70 and 80 cycles/min) were subsequently applied to the animal in every cell tested, with each frequency lasting for 4 min. Spectral analysis revealed that the position of the VO peak in the power spectra altered immediately and correspondingly to the switch of ventilation frequency. Although changes in VO power varied from cell to cell, the alterations in the VO frequency were exactly the same and were consistent with the ventilation frequency in all cells tested (Figure 2A).

Firing rate is an important indicator of neuronal excitability. To figure out whether the sensitivity of a neuron to ventilation is associated with its firing rate, we studied the relationship between the power of VO and the corresponding firing rate. As shown in Figure 2B, there was no significant correlation between the two parameters. In addition, no difference was found between the two categories of GABA neurons (VS and VI) in the firing rate (24.2±1.6 Hz in VS cells vs. 24.1±1.2 Hz in VI cells; *p* = 0.98; Figure 2C) or firing rate distribution (*p* = 0.99; Kolmogorov-Smirnov test).

To directly evaluate the influences of ventilation to the firing, the firing rate was calculated with respect to each pulse of the ventilation. Among the 47 VS GABA neurons studied, 38 (81%) were excited by the ventilation pulse, with the firing rate increased to 159±14.2% of baseline during the pulse. The firing in the other 9 cells (19%), however, was suppressed to 80.4±4.4% of baseline (Figure 2 D and E). It was interesting to note that the strongest excitation or inhibition did not occur at the very end of each pulse. Changes in the firing activity always peaked rapidly upon the arrival of the stimuli and decreased slowly afterwards, indicating a very fast tolerance of GABA neurons to the stimulation. The strongest excitation appeared at 315±13.2 ms after the onset of the pulse (duration of the pulse = 423 ms), while the strongest inhibition was observed at 361±26.2 ms (Figure 2E).

### Most DA Neurons are Inhibited by the Ventilation

**Figure 3:**
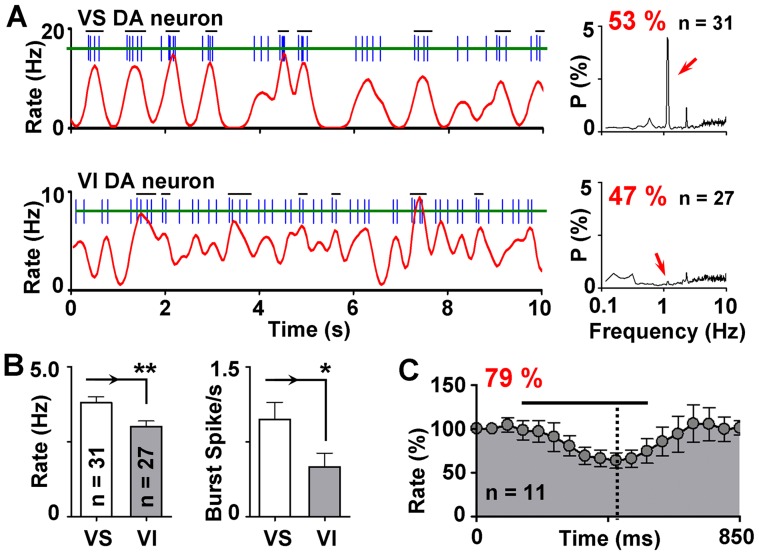
Influences of ventilation on DA neuron firing. (**A**) Schematic drawing of segments of spike trains from a VS DA neuron (top) and a VI DA neuron (bottom) and the averaged power spectra of the two groups of cells. Bursts, defined by the “80/160 ms” criterion, were marked by the horizontal bars above the spike trains. (**B**) Bar graphs showing the differences of firing rate and burst activity between ventilation-sensitive (VS) and ventilation-insensitive VI DA cells. *p<0.05; **p<0.01. (**C**) Averaged changes of the firing rate in response to the ventilation pulse (black line). Dashed line indicates the strongest inhibition point. Most DA neurons were inhibited by the ventilation pulse.

Given that GABA neurons in this region are fundamental regulators of DA cells, the rhythmic activity of VTA GABA neurons may contribute to the activity of DA neurons, and therefore produce a similar VO in DA neurons. To test the hypothesis, we recorded single-unit firing in VTA DA neurons and analyzed the data using the same methods. Consistent with the view, the firing in 53% (31 of 58 cells) of VTA DA neurons also exhibited a prominent VO in their power spectra (Figure 3A). However, different from the GABA neurons that were similar in the firing rate between VS and VI neurons, the firing of VS DA neurons were faster than VI DA neurons (3.8±0.20 Hz in VS DA neurons vs. 3.0±0.19 Hz in VI DA neurons; *p*<0.01). In addition, the burst activity in VS DA neurons was also much stronger than the VI DA neurons (Burst Spike/s = 0.98±0.17 in VS DA neurons vs. 0.49±0.14 in VI DA neurons; *p*<0.05; Figure 3B).

We also analyzed the direct influences of the ventilation pulse to the activity of DA neurons. However, because the firing of VTA DA neurons was very slow, it was hard to evaluate the tendency of firing rate precisely, especially when the VO was not very strong. Therefore we only focused on 14 selected DA neurons with robust VO in this part. The results showed that most DA cells (79%; 11 of 14 cells) were inhibited by the ventilation pulse, with the firing rate decreased to 75.7±8.7% of baseline (Figure 3C). The strongest inhibition occurred at 381±20.1 ms after the onset of the ventilation pulse, which was significantly slower than the strongest excitation in GABA neurons (*p*<0.01), indicating that GABA neurons respond earlier to the stimuli. The other 3 VS DA cells were excited by the ventilation (firing rate = 181±26% of baseline), with the strongest excitation appeared at 351±57 ms after the onset of the stimuli.

### Somatosensory Pathways of the VO

**Figure 4:**
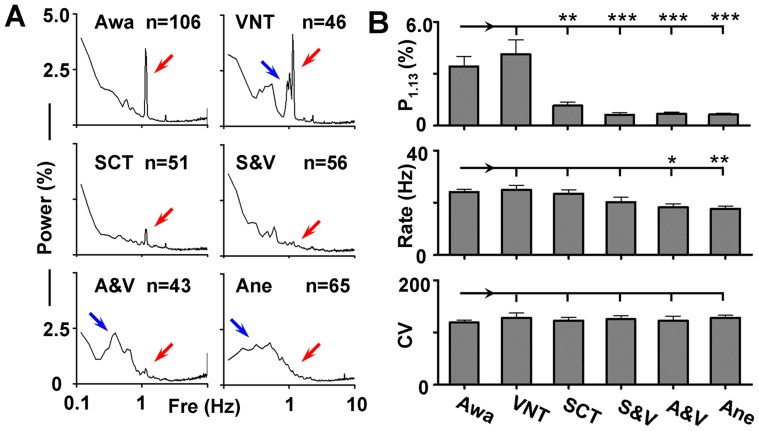
Dependence of VO on sensory pathways and consciousness. (**A**) Averaged power spectra from awake (Awa) rats, rats treated by vagus nerve transection (VNT), spinal cord transaction (SCT), VNT and SCT (V&S), anesthesia under ventilation (A&V), and anesthesia (Ane). Red arrows indicate the position of 1.13 Hz in the spectra, and blue arrows indicate multi-peaks around the VO in VNT neurons and slow oscillation under anesthesia. (**B**) Bar graphs showing the peak power in 1.13 Hz (P_1.13_), firing rate, and coefficient of variation (CV) of the inter-spike intervals under different procedures. *p<0.05; **p<0.01; ***p<0.001 (one-way ANOVA followed by *post hoc* Tukey test, compared with Awa group).

In an effort to identify the peripheral sensory pathways mediating the VO, bilateral vagus nerves were transected in 6 rats. However, no significant change was observed in the VO power of GABA neurons between control (Awa; P_1.13_ = 3.7±0.57; n = 106; P_1.13_ indicates the peak power in 1.13 Hz in a power spectrum and is used to evaluate the intensity of VO in this study) and vagus nerve transected rats (VNT; P_1.13_ = 4.1±0.84; n = 46; *p* = 0.71). Although vagus nerve transection failed to alter the amplitude of the VO, it significantly attenuated the precision of GABA neurons in responding to the ventilation. The power spectrum from rats with vagus nerve transacted exhibited several peaks near the VO peak, which was distingct to only one prominent oscillation in the control group (Awa; Figure 4A). These results indicate that the vagus nerve may not be involved in transmitting the strength of the input, but could actively participate in controlling the precision of the regulation. On the other hand, when spinal cord was transected the VO almost completely disappeared (SCT; P_1.13_ = 1.2±0.18; n = 51; *p<*0.01; compared with Awa group), suggesting that the sensory stimulation is mostly delivered into the brain through the spinal cord. We also recorded the firing patterns of GABA neurons from rats (n = 7) whose spinal cord and vagus nerves were both transected. As expected, no oscillation was seen in the VO frequency band (SCT; P_1.13_ = 0.63±0.11; n = 56; *p<*0.001; compared with Awa group).

Somatosensory-evoked discharges are typically attenuated during anesthesia. For DA cells in the VTA, they can only respond to very strong stimuli under anesthesia [7], [25]. To test if it is also the case for the VO in GABA neurons, rats were anesthetized by chloral hydrate (400 mg/Kg; n = 6) under artificial ventilation. As shown in Figure 4A, the VO peak did not show up under anesthesia (A&V; P_1.13_ = 0.68±0.09; n = 43; *p<*0.001; compared with Awa group). Instead, a prominent slow oscillation could be found in the averaged power spectrum. The firing patterns of neurons from rats under anesthesia were also studied (n = 6). In agreement with previous reports [20], [24], the firing of GABA neurons exhibited a slow oscillation in the mean power spectrum, and no significant activity was found near the frequency of ventilation (Ane; P_1.13_ = 0.64±0.06; n = 43; *p<*0.001; compared with Awa group).

Although corresponding changes in the firing patterns were observed by the manipulations, only anesthesia caused a small but significant reduction in the firing rate. Other procedures (vagus nerve or spinal cord transection), however, induced no alteration in either the firing rate or the mean coefficient of variation (CV) of the inter-spike intervals (Figure 4B and Table 1).

**Table 1:** Firing properties of GABA neurons under denoted conditions.

Groups	n	P1.13	Firing Rate (Hz)	CV
**Awa**	106	3.7±0.57	24.2±0.95	119±4.5
**VNT**	46	4.1±0.84	24.9±1.75	127±9.4
**SCT**	51	1.2±0.18***	23.4±1.39	123±5.9
**S&V**	56	0.63±0.11***	20.3±1.78	125±6.7
**A&V**	43	0.68±0.09***	18.2±1.41*	122±8.7
**Ane**	65	0.64±0.06***	17.7±1.01**	127±4.9

P_1.13_ indicates the peak power at 1.13 Hz, CV indicates the mean coefficient of the inter-spike intervals; VNT indicates vagus nerve transaction; SCT indicates spinal cord transaction; V&S indicates VNT and SCT; A&V indicates anesthesia under ventilation; Ane means anesthesia.

* *p*<0.05;

** *p*<0.01;

*** *p*<0.001 (one-way ANOVA followed by *post hoc* Tukey test, compared with Awa group).

### Roles of N-methyl-D-aspartate (NMDA) Receptor in the VO

**Figure 5:**
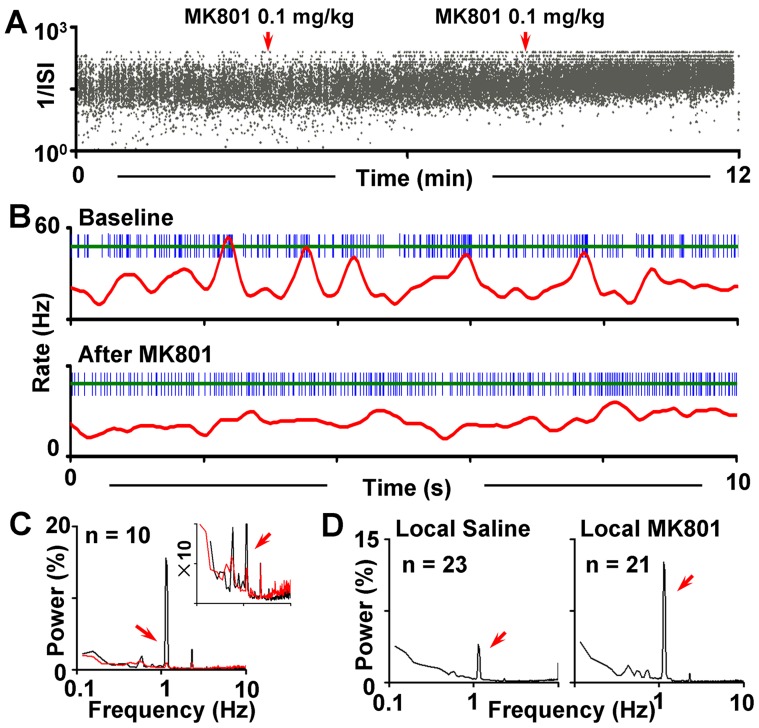
Effects of MK-801 on the VO. (**A**). Plots of inter-spike intervals of a GABA neuron against time, showing that systemic administration of MK-801 gradually changed the distribution of inter-spike intervals from bimodal (two bands) to unimodal (one band), which indicates alterations in the firing pattern. The red arrows indicate the time point when MK-801 was given. The cumulative dosage of the drug was 0.2 mg/kg at the end of the experiment. (**B**) Representative firing patterns of a VS GABA neuron before (top) and after (bottom) the administration of MK-801. (**C**) Superimposed mean power spectra showing the firing patterns before (black) and after the administration of MK-801 (red). The inset is the same spectra displayed on an expanded y-axis scale. (**D**) Mean power spectra of firing patterns of GABA neurons under local MK-801 injection and local saline injection.

To elucidate possible roles of NMDA receptors in modulating the VO, 10 VS GABA neurons were selected. After recording a stable baseline for at least 4 min, MK-801 was delivered gradually (0.2 mg/kg; iv.). Only one cell was studied in each rat. A robust VO inhibition effect was observed by MK-801 administration, with the VO-power decreased from 15.6±4.5 to 0.87±0.21 (*p*<0.001; n = 10; Figure 5). The CV of the firing also showed a significant decrease by the drug (from 117±7.7 in baseline to 97±12 after MK-801; *p*<0.05), indicating a more regular firing pattern after blocking the NMDA receptors. On the other hand, different from those reported in anesthetized animals [16], systemic blockade of NMDA receptors in conscious animals caused varied responses in the firing rate of GABA neurons: 4 of them showed a decrease while the others exhibited an increase (n = 3) or no significant change (n = 3). When averaged together, however, systemic MK-801 administration caused no significant difference in the firing rate (from 18.6±3.0 Hz in baseline to 20.1±3.6 Hz after MK-801 administration; *p* = 0.26).

We next tested whether and how local NMDA receptor located in the VTA participated in the generation of VO. MK-801 was microinjected into the VTA prior to the recording (150 ng in 0.5 µl normal saline), and the firing of GABA neurons were gathered within 60 min after the drug delivery. In contrast to that of systemic MK-801 administration, local NMDA receptor blockade dramatically enhanced the VO (P_1.33_ = 12.6±3.5 in MK-801 treated group, n = 21 vs. 3.9±1.1 in saline-treated group (0.9%, NaCl), n = 23; *p*<0.01; Figure 5D). However, the firing rate was not altered (21.8±3.3 in MK-801 treated group vs. 23.6±2.7 in saline-treated group; *p = *0.66), and so was the CV of inter-spike interval (123±7.1 in MK-801 treated group vs. 132±13.9 in control group; *p = *0.55).

## Discussion

The present study demonstrates that the activity of a proportion of GABA neurons in the VTA in conscious animals can be precisely regulated by aversive sensory input. The regulation depends on peripheral neuronal pathways and consciousness. The study further demonstrates that NMDA receptors play a crucial role in regulating the sensitivity of these neurons to the peripheral stimuli. Moreover, by recording single DA neuron firing, this work also implies that the responses of DA neurons to aversive sensory input may be mediated by these GABA neurons.

The VTA is a heterogeneous brain area containing several neuronal populations including DA, GABA and glutamatergic neurons [26]. The mixture of these neurons acts in concert to orchestrate the output of VTA, which will contribute to reward-seeking behaviors, as well as attentional or alerting reactions [6], [18]. The inhibitory network that regulates DA neuron activity is increasingly well described. In our study, a correlation between GABA cell firing and ventilation was evident when the rate histograms and the power spectra were directly compared with the pressure wave of the ventilation. Although not as noxious as tail pinch or electric shock, the pressure pulses generated by the ventilation could serve as mild aversive stimuli as most DA neurons were inhibited by the ventilation pulse [7], [27]. Since local GABA neurons are primary inhibitory components for DA neurons, our results may explain why most DA neurons are inhibited by aversive stimuli [7]: During aversive stimuli, the activity of DA neurons may be inhibited indirectly via local GABA neurons which are activated by the stimuli. In support of this view, the DA neurons under our condition were also sensitive to the stimuli, and exhibited nearly antiphase relationship with GABA neurons. This is further supported by the fact that responses of GABA neurons to the ventilation pulse was ahead of DA neurons (Figure 2 & 3). Our observation is in agreement with a very recent study showing that local GABA neurons activated by aversive stimuli such as footshock are indeed able to inhibit the DA cell firing and produce alterations in behavior [19].

It was noted that although most GABA neurons were excited by the ventilation, there were still neurons showing inhibition, indicating a heterogeneous populations of GABA neurons in the VTA. The GABA neurons in the VTA receive inhibitory afferents from medium spiny neurons of the nucleus accumbens, as well as excitatory projections from the prefrontal cortex and lateral habenula [28], [29], [30]. The activity of VTA GABA neurons are determined by the dynamic balance of these inputs. Due to varied projection or functional regulations, the firing pattern of the neurons can be regulated differently. Consistently, previous studies have indeed identified several GABA subpopulations in the VTA according to their distinct projection properties [31], [32]. In addition, studies have also indicated that DA neurons in the VTA are anatomically and functionally subdivided [27]. It is thus possible that the GABA neurons in this area exhibit a similar distribution pattern. Although the firing pattern of GABA neurons is very sensitive to manipulations of sensory pathways, the firing rate is quite resistant. This could be due to the fact that VTA GABA neurons are interconnected both by chemical synapses and by electrical synapses or gap-junctions [33]. The responses to peripheral stimuli may be resulted from chemical input, whereas the basic activity may be determined by gap-junctions. Therefore, the sensory input mediated by chemical input will produce little influence to the firing rate. On the other hand, both the present work and previous studies demonstrate that the firing rate was depressed by chloral hydrate [24], most probably because of the overall decrease in the excitability of the brain.

Although further investigations are still required to identify the mechanisms and upstream structures that drive the GABA neuronal response to aversive stimuli, our work demonstrated that systemic NMDA receptor blockade completely disrupted the response of GABA neurons to the peripheral stimuli. The result is of particular interest, as MK-801 treatment is known to mimic schizophrenia-like behaviors [34]. The loss of sensitivity in GABA neurons to sensory stimuli due to NMDA antagonist administration may imply a potential important role of VTA GABA neurons in mediating schizophrenic syndromes. It is noted that previous report has showed that NMDA receptor blockade reduced the GABA neuronal firing rate in anesthetized animals [16]. However, systemic MK-801 administration caused various effects in our study. The differences are most likely attributed to the fact that the data in the present study were recorded from conscious animals. Another interesting observation is that local NMDA blockade specifically enhanced the sensitivity of GABA neurons to the sensory stimuli, leaving other firing parameters intact. The elevated sensitivity might be due to enhanced input or coupling of the local GABA neurons or other complex network interactions, given the fact that the VTA GABA neurons receive several forms of input from other brain regions [28], [29], [30]. Further investigations, however, are still required to elucidate the paradoxical responses.
